# The Association of Life’s Simple 7 with Aldosterone among African Americans in the Jackson Heart Study

**DOI:** 10.3390/nu11050955

**Published:** 2019-04-26

**Authors:** Veena Kesireddy, Yubo Tan, David Kline, Guy Brock, James B. Odei, Bjorn Kluwe, Valery S. Effoe, Justin B. Echouffo Tcheugui, Rita R. Kalyani, Mario Sims, Herman A. Taylor, Morgana Mongraw-Chaffin, Ehimare Akhabue, Joshua J. Joseph

**Affiliations:** 1 Department of Medicine, The Ohio State University Wexner Medical Center, Columbus, OH 43210, USA; veena.kesireddy@osumc.edu (V.K.); kluwe.3@buckeyemail.osu.edu (B.K.); 2 Department of Biomedical Informatics and Center for Biostatistics, The Ohio State University Wexner Medical Center, Columbus, OH 43210, USA; yubo.tan@osumc.edu (Y.T.); david.kline@osumc.edu (D.K.); guy.brock@osumc.edu (G.B.); 3 Division of Biostatistics, College of Public Health, The Ohio State University, Columbus, OH 43210, USA; odei@stat.osu.edu; 4 Department of Medicine, Morehouse School of Medicine, Atlanta, GA 30310, USA; VEffoe@msm.edu (V.S.E.); htaylor@msm.edu (H.A.T.); 5 Division of Endocrinology, Diabetes, and Metabolism, Johns Hopkins University School of Medicine, Baltimore, MD 21287, USA; jechouf1@jhmi.edu (J.B.E.T.); rita.kalyani@gmail.com (R.R.K.); 6 Department of Medicine, University of Mississippi Medical Center, Jackson, MS 39216, USA; msims2@umc.edu; 7 Department of Epidemiology & Prevention, Wake Forest School of Medicine, Winston-Salem, NC 27157, USA; mmongraw@wakehealth.edu; 8 Division of Cardiovascular Diseases and Hypertension, Rutgers University Robert Wood Johnson Medical School, New Brunswick, NJ 08901, USA; ehimare.akhabue@rutgers.edu

**Keywords:** aldosterone, ideal cardiovascular health, Life’s Simple 7, African Americans, physical activity, body mass index, smoking, blood pressure, glucose, cholesterol.

## Abstract

Background: Among African Americans (AAs), attaining higher levels of American Heart Association (AHA) ideal cardiovascular health (Life’s Simple 7 [LS7]) is associated with lower risk of diabetes and cardiovascular disease (CVD). We previously showed that aldosterone is associated with higher risk of diabetes and CVD in AAs. Thus, we investigated the association of LS7 metrics with aldosterone in the Jackson Heart Study (JHS). Methods: Ideal metrics were defined by AHA 2020 goals for health behaviors (smoking, dietary intake, physical activity, and body mass index) and health factors (total cholesterol, blood pressure, and fasting glucose). The number of ideal LS7 metrics attained at baseline were summed into a continuous score (0–7) and categorical groups (Poor: 0–1, Intermediate: 2–3, and Ideal: ≥4 ideal LS7 metrics). Multivariable linear regression was used. Results: Among 4,095 JHS participants (mean age 55 ± 13 years, 65% female), median serum aldosterone was 4.90, 4.30, and 3.70 ng/dL in the poor (*n* = 1132), intermediate (*n* = 2288) and ideal (*n* = 675) categories respectively. Aldosterone was 15% [0.85 (0.80, 0.90)] and 33% [0.67 (0.61, 0.75)] lower in the intermediate and ideal LS7 categories compared to the poor LS7 category. Each additional LS7 metric attained on continuous LS7 score (0–7) was associated with an 11% [0.89 (0.86, 0.91)] lower aldosterone level with variation by sex with women having a 15% lower aldosterone vs. 5% in men. Conclusions: Higher attainment of ideal LS7 metrics was associated with lower serum aldosterone among AAs with a greater magnitude of association among women compared to men.

## 1. Introduction

In 2010, the American Heart Association (AHA) designed and promoted a set of seven cardiovascular health (CVH) metrics (Life’s Simple 7 [LS7]) aimed at reducing cardiovascular disease (CVD) [1]. The metrics are divided into four health behaviors (smoking, weight, physical activity, and diet) and three health factors (blood pressure, total cholesterol, and glucose). Higher attainment of ideal LS7 metrics is associated with a lower risk of incident type 2 diabetes (diabetes), CVD and all-cause mortality [2,3,4]. There is currently limited exploration of LS7 with biological hormones that contribute to adverse cardiometabolic outcomes. Higher aldosterone has been associated with higher incidence of metabolic syndrome, diabetes, CVD and all-cause mortality among African Americans (AA) and thus represents one such potential hormone [5,6,7]. Additionally, there is limited data on the association of the individual LS7 metrics with aldosterone among AAs. Previously, studies have shown positive associations of blood pressure (BP) [7,8], fasting plasma glucose (FPG) [5], body mass index (BMI) [8], and cholesterol with aldosterone in majority non-Hispanic white (NHW) studies [8,9,10,11,12]. There is a paucity of data regarding the association of smoking, physical activity (PA), and diet with aldosterone and no studies assessing the association of the combination of modifiable risk factors assessed by the LS7 with aldosterone in any racial/ethnic group. Given the higher prevalence of diabetes, CVD and mortality among AAs compared to NHWs [3], it is critical to determine the relationship of LS7 with biological hormones important in cardiometabolic disease. Thus, the goal of this study was to examine the association of LS7 with aldosterone, characterizing the association of combined metrics of LS7 with aldosterone as our primary outcome and the association of individual metrics of LS7 with aldosterone as our secondary outcomes. We hypothesized an inverse association of attainment of LS7 metrics with serum aldosterone.

## 2. Methods

### 2.1. Study Participants

The Jackson Heart Study (JHS) is a prospective study of the development and progression of CVD in a cohort of 5306 AA adults, aged 21–94 years. Briefly, participants were enrolled from the three counties that make up the Jackson, Mississippi metropolitan area. Participants provided extensive medical and sociodemographic information, and had physical and biochemical measurements. Enrollment and baseline examinations were performed from 2000–2004. Participants were excluded for missing the following data at baseline: LS7 metrics (*n* = 1186), aldosterone (*n* = 113), and covariates (*n* = 147). Excluded vs. included participants had a higher percentage of men, smoking, diabetes, glycosylated hemoglobin A1c (A1c) with lower educational attainment, professional occupations, diastolic blood pressure (DBP), and total cholesterol with no difference in aldosterone (Table S1). After these exclusions, 4095 participants were included in this cross-sectional analysis. Details about the study design, recruitment, and methods used have been described elsewhere [13]. The JHS was approved by the institutional review boards of the University of Mississippi Medical Center, Jackson State University, and Tougaloo College. All participants provided informed consent. 

### 2.2. Exposures

The primary exposure was ideal CVH assessed using the LS7 metrics including cigarette smoking status, diet, physical activity, BMI, total cholesterol, FPG, and BP. Each baseline metric was evaluated separately using poor, intermediate, and ideal categories as defined by AHA guidelines (Table S2) [2]. We assigned participants one point per LS7 metric in the ideal category and zero points for intermediate or poor categories [2].

#### 2.2.1. Ascertainment of Life’s Simple 7 Metrics

##### Blood Pressure

Blood pressure was measured twice at 5-minute intervals by using Hawksley random zero sphygmomanometers in the sitting position and averaged for analyses. Ideal blood pressure was defined as untreated systolic blood pressure (SBP) < 120 mmHg and diastolic blood pressure (DBP) < 80 mmHg; intermediate blood pressure was defined as systolic blood pressure of 120–139 mmHg or diastolic blood pressure of 80–89 mmHg, or if treated to goal (<120/<80 mmHg); and poor BP was defined as systolic blood pressure of ≥140 mmHg or a diastolic blood pressure ≥ 90 mmHg [1,2].

##### Physical Activity

PA was assessed using an interviewer-administered PA questionnaire at baseline, modified from the Baeke PA survey [14]. This instrument was identical to the one used during the Kaiser Physical Activity survey, which showed good validity and reliability in a multiethnic sample [15]. Exercise was reported by the average amount of time per week spent, and metabolic equivalent levels were defined for each activity. Moderate activity was defined as 3–6 metabolic equivalents and vigorous activity as >6 metabolic equivalents. Ideal PA was defined as ≥150 min of moderate-intensity activity or ≥75 min of vigorous activity or ≥150 min of combined moderate-intensity and vigorous activity per week; intermediate PA was defined as 1–149 min of moderate-intensity activity or 1–74 min of vigorous activity or 1–149 min of combined moderate-intensity and vigorous activity per week; poor PA was defined as no amount of activity (0 min per week) [1]. 

##### Body Mass Index

BMI was calculated by dividing weight (kilograms) by the square of height (meters). BMI < 25 kg/m^2^ was considered ideal; 25–29.9 kg/m^2^ was intermediate; and ≥30 kg/m^2^ was poor [1]. 

##### Smoking

Cigarette smoking was self-reported, and participants were asked for quantity and duration of smoking. Participants who had never smoked or who quit smoking more than 12 months preceding the clinic visit were considered ideal; Former smokers who had quit smoking within 12 months of the clinic visit were considered intermediate; and current smokers were considered poor [1]. 

##### Total Cholesterol

Total cholesterol was measured by the cholesterol oxidase method (Roche COBAS Fara analyzer; Roche Diagnostics), as previously described [3]. Ideal total cholesterol was defined as an untreated total cholesterol <200 mg/dL; intermediate total cholesterol defined by a level of 200–239 mg/dL or if treated to goal (<200 mg/dL); and poor was defined by a level of ≥240 mg/dL [1]. 

##### Diet

In the JHS, dietary intake was assessed using the Delta Nutrition Intervention Research Initiative food frequency questionnaire with 158 items [16]. The questionnaire had some slight differences compared with the AHA categorization regarding units of servings, requiring modification of the metrics, as has been done previously [1,3]. The five dietary components used to compute the AHA score, based on a 2000-kcal diet, were: (1) fruits and vegetables of 4.5 cups/day or more, (2) two 3.5-oz servings or more per week of non-fried fish, (3) fiber-rich whole grains of three 1-oz-equivalent servings/day or more, (4) sodium less than 1500 mg/day, and (5) sugar sweetened beverages of 450 kcal/week or less (36 oz.). Ideal diet was defined by a diet including 4–5 components; intermediate diet, 2–3 components; and poor diet, 0–1 component [1]. 

##### Glucose

At baseline, fasting plasma glucose was measured by the glucose oxidase colorimetric method using a Vitros 950 or 250 (Ortho Clinical Diagnostics analyzer; Ortho Clinical Diagnostics, Raritan, NJ, USA). Ideal glucose was defined as an untreated fasting plasma glucose of <100 mg/dL; intermediate glucose was defined as a fasting plasma glucose of 100–125 mg/dL untreated or if treated to goal (<100 mg/dL); and poor was defined as a fasting plasma glucose of ≥126 mg/dL [1].

#### 2.2.2. Life’s Simple 7 Score

Categorical scores were calculated by summing the number of ideal LS7 metrics each participant attained. We then categorized each participant into poor (0–1 ideal LS7 metrics), intermediate (2–3 ideal LS7 metrics) or ideal (4+ ideal LS7 metrics) LS7 score categories. Continuous scores were calculated based on the number of ideal LS7 metrics each participant attained with the score ranging from 0–7 [2]. We performed a sensitivity analysis, using the total LS7 score ranging from 0–14, calculated as the sum of the LS7 metric scores. This score was classified into three levels as inadequate (0–4), average (5–9), or optimal (10–14) LS7 [2].

### 2.3. Outcome

#### 2.3.1. Serum Aldosterone

Fasting blood samples were drawn in the supine position and processed using a standardized protocol. Plasma and serum were prepared from samples by sedimentation in a refrigerated centrifuge within two hours of blood collection, stored at −70 °C and sent to the central laboratories (University of Minnesota) [3,13]. Lower limit of serum aldosterone value was 1.9 ng/dL and approximately 15% had levels below 1.9 ng/dL. Serum aldosterone was measured by radioimmunoassay (Siemens) and the intra-assay coefficients of variation were 8.7% for low and 6.2% for high concentrations.

#### 2.3.2. Covariates

Baseline information was obtained during clinic visits or at home using standardized questionnaires including: demographics (age, sex), occupation (management/professional versus not), level of education (≥Bachelor’s degree versus <Bachelor’s degree), alcohol use (in the past 12 months versus not), and current prescription medication usage. Estimated glomerular filtration rate (eGFR) was derived using the Chronic Kidney Disease Epidemiology Collaboration equation (mL/min/1.73 m^2^) [17]. EGFR was included as a covariate due to a known inverse association with aldosterone [17]. Sodium, potassium, creatinine, and chloride were measured on a Vitros 950 or 250, Ortho-Clinical Diagnostics analyzer (Raritan, NJ, USA) at the University of Mississippi Medical Center [13,18]. A1c concentrations were measured using a high-performance liquid chromatography system (Tosoh Corp, Tokyo, Japan) [18].

### 2.4. Statistical Analysis

Baseline characteristics of participants are presented by poor, intermediate, and ideal levels of LS7 score using one-way analysis of variance for normally distributed continuous variables, Kruskal–Wallis test for non-normally distributed continuous variables and the Chi-square test for categorical variables. We performed the Cochran–Armitage Trend test for median aldosterone levels per LS7 score category. Due to a right skewed distribution, serum aldosterone was log-transformed for analyses. Log-aldosterone was modeled by categorical and continuous LS7 scores respectively, using multivariable linear regression with adjustments for age, sex, education, occupation, alcohol use, and eGFR. For continuous LS7 scores, the effect of a one-unit change in the composite score was evaluated. For categorical LS7 score, serum aldosterone in the intermediate and ideal categories was compared to the poor category. We performed similar analyses for the individual LS7 metrics. The linear regression model is fit on the log scale and the mean differences are on the log scale. As the log scale is not clinically interpretable, we back-transformed estimates to the original aldosterone scale for interpretation. On the original scale, the estimates provide the ratio of the means between the two groups being compared, and thus the mean ratios are presented [19]. Based on the ratio, one can make a relative comparison in terms of percent change. Covariates were selected a priori with adjustment for age, sex, education, occupation, alcohol use, and eGFR based on prior analyses [2,5,6].

Given that the association of aldosterone with LS7 may vary by age, sex, diabetes status, and eGFR, we tested for effect modification of these factors with LS7 by inserting an interaction term in the model and used the likelihood ratio test. Diabetes was defined as A1c ≥ 6.5% (48 mmol/mol), fasting blood glucose ≥126 mg/dL, taking diabetes medications or a self-reported physician diagnosis. We performed sensitivity analyses excluding participants on medications that alter aldosterone including angiotensin converting enzyme inhibitors, angiotensin receptor blockers, mineralocorticoid receptor antagonists, and statin medications [6]. Aldosterone initially decreases with ACE-inhibitors and angiotensin receptor blockers but over time aldosterone levels increase to pretreatment levels or above, a phenomenon termed aldosterone breakthrough. Mineralocorticoid receptor antagonists increase aldosterone, whereas statins decrease aldosterone [20]. To confirm the robustness of our findings, we excluded individuals with aldosterone below the lower detection limit and performed the primary analyses stated above, (Table S3). Statistical significance was defined as two-sided alpha < 0.05 and *p* < 0.10 for interaction terms. Given the exploratory nature of the analyses, the results were reported at a nominal level. Analyses were performed using SAS 9.4 (SAS Institute Inc., Cary, NC, USA).

## 3. Results

Among 4095 adults (mean age 55 ± 13 years, 65% female), there were 1132 (28%) participants with poor LS7 scores, 2288 (56%) with intermediate LS7 scores and 675 (16%) with ideal LS7 scores (Table 1). Participants in the ideal LS7 score category had a more optimal cardiovascular profile with lower glucose, SBP, DBP, BMI, total cholesterol and higher levels of educational attainment, professional occupational status, and eGFR compared to intermediate and poor categories (*p*-value for comparisons < 0.0001). Median serum aldosterone was 4.90, 4.30, and 3.70 ng/dL in the poor, intermediate and ideal categories, respectively [*p* for trend = 0.004]. Serum potassium, chloride and potassium intake were modestly higher, whereas serum sodium and creatinine were modestly lower across categories of poor, intermediate, and ideal LS7.

Median serum aldosterone levels stratified by age, sex, diabetes status, and eGFR are shown in Table 2. Median aldosterone was higher in men (4.80 ng/dL) compared to women (4.10 ng/dL), in participants with diabetes (4.70 ng/dL) compared to participants without diabetes (4.20 ng/dL) and in participants with eGFR below median (5.00 ng/dL) compared to participants with eGFR above median (3.80 ng/dL) [*p* < 0.0001 for all comparisons]. The findings remained significant after exclusion of participants taking medications that alter aldosterone (Table 3). Sensitivity analysis conducted using the total LS7 score revealed similar findings for median aldosterone (Table S4).

The associations of the categorical and continuous LS7 scores with aldosterone are shown in Table 2. Aldosterone in the ideal and intermediate categories of LS7 score was 33% (Mean Ratio: 0.67, 95%CI: 0.61, 0.75) and 15% (Mean Ratio: 0.85, 95%CI: 0.80, 0.90) lower compared to the poor category. For continuous LS7 score, each additional ideal LS7 metric attained [a one-unit increase in score (0–7)] was associated with 11% (Mean Ratio: 0.89, 95%CI 0.86, 0.91) lower aldosterone, with similar estimates after adjustment for aldosterone altering medications. Sensitivity analysis conducted using the total LS7 score revealed significant mean ratios (Table S4). Additionally, excluding participants taking medications that alter aldosterone revealed similar findings where aldosterone in the ideal and intermediate categories of LS7 score was 38% (Mean Ratio: 0.62, 95%CI: 0.55, 0.70) and 27% (Mean Ratio: 0.73, 95%CI: 0.67, 0.79) lower compared to the poor category (Table 3). For continuous LS7 score, each additional ideal LS7 metric attained [a one-unit increase in score (0–7)] was associated with a 14% (Mean Ratio: 0.86, 95%CI 0.83, 0.89) lower aldosterone.

Effect modification of the association of LS7 with aldosterone was significant for age (*p* < 0.001), sex (*p* < 0.001 for continuous and *p* = 0.0021 for categorical LS7 scores), eGFR (*p* < 0.001) and diabetes (*p* = 0.016 for categorical LS7 score) among all participants (Table 2). Sex stratified analyses among all participants (Table 2) revealed that women had 39% (Mean Ratio 0.61, 95% CI: 0.53, 0.70) whereas men had 19% (Mean Ratio 0.81, 95% CI: 0.69, 0.96) lower aldosterone in ideal vs. poor LS7 score categories. For continuous LS7 score association with aldosterone, for each additional LS7 metric attained, women had 15% lower aldosterone (Mean Ratio: 0.85, 95% CI: 0.82, 0.88) vs. 5% lower aldosterone (Mean Ratio 0.95, 95% CI: 0.91, 0.98) in men. These findings remained significant after excluding participants taking aldosterone altering medications (Table 3), whereas findings for age, eGFR and diabetes status were attenuated and non-significant after excluding individuals taking aldosterone altering medications (Table 2 and Table 3). Excluding individuals below the lower detection limit of the aldosterone assay did not alter the significance of our findings (Table S3).

Among the individual LS7 metrics (Table 4), ideal categories of smoking, BMI, blood pressure, total cholesterol, and fasting plasma glucose were associated with lower aldosterone compared to poor categories (all comparisons *p* < 0.05). The intermediate vs. poor categories of individual LS7 metrics were significant for fasting plasma glucose (*p* < 0.0001). There was significant effect modification by sex, and in stratified models, generally a greater magnitude of mean ratios for the ideal vs. poor category comparisons for women as compared to men, except for BMI. Notably, the greatest difference was for ideal vs. poor categories of smoking with 4% lower aldosterone in men (mean ratio 0.96, 95% CI: 0.83, 1.10) vs. 42% lower aldosterone in women (mean ratio 0.58, 95% CI: 0.53, 0.64), which remained significant in sensitivity analysis excluding individuals taking aldosterone altering medications (Table S5).

## 4. Discussion

The JHS is an AA community-based cohort study with well-characterized data on LS7 metrics and aldosterone. This first report of the association of LS7 with aldosterone demonstrates a strong inverse association of LS7 scores with aldosterone, with a greater magnitude of effect among women. Among the individual metrics attaining ideal vs. poor categories of glucose, blood pressure, BMI, smoking status, and total cholesterol were associated with lower aldosterone, with a generally greater magnitude in women except for BMI. This is also the first study to reveal higher serum aldosterone in AA men compared to women, independent of medications that alter aldosterone. 

### 4.1. The Association of LS7 Score with Aldosterone

Although the individual metrics of LS7 have been associated with lower long-term CV risk in multiple studies, the biological underpinnings remain understudied. Here we show that more optimal levels of five of the seven metrics and the overall score are significantly associated with lower aldosterone, independent of aldosterone altering medications. We are aware of no other studies examining the association of similar combinations of lifestyle modifiable risk factors with aldosterone. Although the difference between the aldosterone values is small (highest to lowest 1.20 ng/dL), prior data revealed significant increases in the risk of diabetes with similar changes in aldosterone emphasizing the biological significance [5]. Racial differences in aldosterone were noted previously with lower aldosterone levels among AAs compared to NHWs, Hispanics, and Chinese Americans [21]. However, this study reveals novel sex differences with higher serum aldosterone among men, but a greater magnitude of association of higher LS7 score with lower aldosterone among women. We initially hypothesized these findings may be secondary to higher aldosterone in women compared to men as had been shown previously among NHWs in the Framingham Heart Study, which collected aldosterone using similar parameters [10]. Discordant with the Framingham Heart Study findings, we found a 17–18% higher aldosterone among men in the JHS independent of aldosterone altering medications. A recent study by Toering et al among whites showed that men on a high-salt diet (4.6 grams/day) had higher serum aldosterone than women, with no aldosterone sex differences on a low-salt diet [22]. In the JHS, the average dietary sodium intake was 3.5 grams per day [5], much closer to the high-salt diet in the study, and thus our finding of higher aldosterone in men is consistent with this study, but does not explain the greater magnitude of association of higher LS7 score with lower aldosterone among women. Toering et al also evaluated the adrenal response to exogenous angiotensin II which was significantly higher in women [22]. Thus, there may be sex differences in the regulation of aldosterone with greater biological adrenal responsiveness among women. This hypothesis is supported by a recent finding of 15% greater aldosterone response to Ang II on both restricted and liberal salt diets among women in the majority NHW Hypertensive Pathotype (HyperPATH) consortium [23]. In a rodent model of aldosterone-mediated cardiovascular disease, myocardial damage and proteinuria were greater in female rats despite having similar blood pressure responses [23]. Thus, our finding of sexual dimorphism in relationship of LS7 score with aldosterone is potentially secondary to greater responsivity of aldosterone to endogenous aldosterone agonists in women. 

### 4.2. The Association of Individual LS7 Health Factors and Health Behaviors with Aldosterone: Human Studies and Mechanisms

#### 4.2.1. Cholesterol

Total cholesterol is the overall amount of cholesterol in the blood including a combination of high-density lipoprotein (HDL), low-density lipoprotein (LDL), and triglycerides, calculated as HDL + LDL + (triglycerides/5). There is limited data on the association of aldosterone with total cholesterol. Among majority NHWs, aldosterone has been positively associated with total cholesterol/HDL ratio [10], triglycerides, and inversely associated with HDL [11]. In smaller studies of AA adults, there have been inconsistent findings for the association of cholesterol with aldosterone including positive associations with triglycerides but not total cholesterol [24], positive associations of total cholesterol and triglycerides with standing but not supine aldosterone [8], and an inverse association of HDL with aldosterone with no reported association with total cholesterol or triglycerides [9]. In the JHS, aldosterone was positively associated with change in triglycerides, but not HDL over 4 years with no previous assessment of total cholesterol [7]. From a mechanistic perspective, cholesterol regulates aldosterone synthesis and regulation via various lipoprotein components. Very low-density lipoprotein through various signaling pathways (STaR and aldosterone synthase) induces aldosterone synthesis [12]. LDL provides substrate (cholesterol) for aldosterone synthesis, thus increases aldosterone levels [25]. Aldosterone production in human adrenocortical cells is stimulated by HDL2 through increased expression of aldosterone synthase (*CYP11B2*) [26]. Consistent with regulatory function of cholesterol, statins lower aldosterone levels [20]. Thus, our finding of a positive association of aldosterone with total cholesterol is the first report in a large population of AAs and is supported by mechanistic studies. 

#### 4.2.2. Glucose

In this study, ideal (FPG < 100 mg/dL) and intermediate (FPG 100–125 mg/dL) glucose categories compared to poor (≥126 mg/dL) were associated with lower aldosterone. Aldosterone excess impairs insulin secretion and insulin sensitivity [27]. Aldosterone increases insulin resistance through inhibition of insulin signaling and insulin-stimulated glucose uptake via glut-4 translocation in adipocytes, skeletal muscle, and vascular smooth muscle cells [28]. Additionally, aldosterone impairs adipokines and nuclear receptors that improve insulin sensitivity through adipose tissue inflammation including adiponectin and peroxisome proliferator-activated receptor-gamma [29]. Among AAs in the JHS, aldosterone was positively associated with insulin resistance, glucose, and change in glucose over 4 years, consistent with studies in other racial/ethnic groups [5,7]. 

#### 4.2.3. Blood Pressure

Aldosterone is associated with higher blood pressure, increases in blood pressure over time, and predicts hypertension among AAs [7,8,30] and whites in single race/ethnicity studies [31,32]. In studies including both AAs and whites, the findings have been mixed, correlations of blood pressure with aldosterone have been more consistent and striking in AAs throughout the lifespan with some studies showing no association in whites [30,33]. In the Multi-Ethnic Study of Atherosclerosis, there was no association of aldosterone with blood pressure among NHWs or AAs [21]. Consistent with the majority of studies in AAs, ideal vs. poor blood pressure categories were associated with lower aldosterone in this analysis.

#### 4.2.4. Body Mass Index

Measures of adiposity including BMI, visceral adipose tissue, and waist circumference have been positively associated with aldosterone among NHWs [9,34]. The findings from smaller populations of AA adults support a cross-sectional association of anthropometric measures of adiposity including body mass index, waist circumference and waist:height ratio with aldosterone [8,9]. Among AAs in the JHS, aldosterone was not associated with change in waist circumference over 4 years, but cross-sectional measures have not been assessed [7]. This study reveals a positive association of body mass index with aldosterone, consistent with the findings among NHWs and provides further evidence for a significant relationship among AAs. Potential mechanisms for the association of BMI with aldosterone are adipokines. Higher levels of BMI are associated with higher levels of leptin and lower levels of adiponectin [35]. Leptin is a direct regulator of *CYP11B2* (aldosterone synthase) expression and aldosterone production in adrenal zona glomerulosa cells [36]. Adiponectin on the contrary decreases steroidogenesis including aldosterone production [37]. There is also a reciprocal interaction between aldosterone and adiposity with aldosterone induced mineralocorticoid receptor activation promoting inflammation, adipocyte differentiation, and alteration of adipokine expression (higher leptin/lower adiponectin) leading to further production of aldosterone [38,39]. Additionally, adipocytes possess aldosterone synthase and can produce aldosterone independently and stimulate production of a liver-derived adrenal aldosterone secretagogue [39]. The BMI-aldosterone association among AAs in the JHS suggest that studies showing weight loss lowers aldosterone levels, may also be relevant for AAs [34,40,41].

#### 4.2.5. Physical Activity

Prior studies analyzing the relationship between aldosterone and physical activity have been inconclusive. Some studies reveal that physical activity in the form of aerobic exercise decreases aldosterone levels [42,43], while others have shown no effect of aerobic exercise on aldosterone [44,45]. Interestingly, there may be racial/ethnic differences with one study revealing lower aldosterone after aerobic exercise training among whites but not AAs [44]. Long-term endurance training has not been associated with reductions in aldosterone in the young or elderly [46]. Physical activity was not associated with aldosterone in our study. Further, epidemiologic and clinical trial research with objective measurement of physical activity is needed to improve the understanding of acute and chronic effects of physical activity on aldosterone.

#### 4.2.6. Smoking

Ideal vs. poor smoking had the greatest magnitude of association with lower aldosterone in this study. Previous data on the association of acute and chronic smoking with aldosterone are mixed. One study noted that aldosterone levels rise acutely after smoking with a peak at 30-minutes [47]. Laustiola et al noted that baseline aldosterone levels in chronic smokers are higher compared to non-smoking identical twins [48]. However, some population based studies have not shown significant differences in aldosterone levels between smokers and non-smokers [49]. In this study, notable sex differences were found with a 4% non-significant lower aldosterone in the ideal (non-smoking) compared to poor group (current smoking) among men and 42% significantly lower aldosterone among women. Cigarette smoke contains over 4000 chemicals; one of the major chemicals is nicotine. In cultured human endothelial cells, nicotine increases the expression and activity of angiotensin-converting enzyme [50,51]. Angiotensin converting enzyme converts angiotensin I to angiotensin II and angiotensin II stimulates secretion of aldosterone from the adrenal cortex. Thus, nicotine increasing angiotensin II through higher angiotensin converting enzyme activity leading to higher aldosterone in smokers, particularly women, due to the blunted adrenal response to angiotensin II in men, may provide one explanation for the sex difference in the smoking–aldosterone association [22,23]. Given the immense number of chemicals in cigarettes, further epidemiological and preclinical studies examining the effects of smoking on aldosterone are needed to clarify this relationship and potential sexual dimorphism.

#### 4.2.7. Dietary Intake

The role of dietary intake consistent with the AHA ideal diet, including fruits and vegetables, fish, fiber-rich whole grains, low sodium diet, and limited intake of sugar sweetened beverages has not been examined in relation to aldosterone. Prior studies have shown that low sodium diets increase plasma aldosterone levels [52]. The ideal diet according to AHA LS7 was not associated with lower aldosterone levels in our study. One of the challenges is accurately measuring dietary intake via subjective measures such as food frequency questions. Further studies focusing on dietary intake and aldosterone may be helpful in delineating the relationship. 

### 4.3. Strengths and Limitations

The strengths of our analysis include a large, contemporary, population-based cohort with rigorously ascertained physiologic and laboratory measures. Additionally, the JHS used validated questionnaires and has a comprehensive documentation of medications, which are critical to categorizing participants into the correct LS7 categories. Despite these and other strengths, there are some potential limitations. First, this is a cross-sectional study, so neither temporality nor causation can be ascertained. Second, the self-reported diet and physical activity were not associated with aldosterone potentially due to misclassification across the categories. Also, only 19% and 0.9% achieved ideal levels of physical activity and diet, respectively, which may have limited the ability to detect significant differences. Third, some molecules that may potentially impact with aldosterone were not collected (blood urea nitrogen, plasma and urinary catecholamines) or were not available in the full cohort (plasma renin activity). Fourth, renin–angiotensin–aldosterone medications may have impacted the aldosterone levels. Thus, sensitivity analysis was performed excluding participants on medications that influence aldosterone including ACE-inhibitors, angiotensin receptor blockers, mineralocorticoid receptor antagonists, and statin medications, which revealed persistent associations of LS7 score with aldosterone. Lastly, the participants in the JHS are from one geographic area in the southeastern United States and may not be representative of all AAs.

## 5. Conclusions

The present study demonstrates that ideal and intermediate vs. poor categories of LS7 are associated with lower aldosterone with sexual dimorphism and a greater magnitude in women. Among the individual metrics, attaining ideal vs. poor levels of glucose, blood pressure, BMI, smoking status, and total cholesterol was associated with lower aldosterone. Given the significant association of LS7 metrics with aldosterone among AAs, further studies are indicated to examine the role of aldosterone as a mediator of the association of Life’s Simple 7 with lower risk of cardiovascular disease and diabetes, and potentially to provide a target for prevention of cardiometabolic disease.

## Figures and Tables

**Table 1:** Baseline characteristics.

Variables ^a^	All (*n* = 4095)	Life’s Simple 7			*p*-values ^d^
Mean (SD)/*N* (col%)		Poor (0–1) *n* = 1132	Intermediate (2–3) *n* = 2288	Ideal (4+) *n* = 675	
Age	55.2 (12.6)	59.9 (10.7)	55.5 (12.0)	46.3 (12.7)	<0.0001
Sex					
Men	1451 (35.43)	389 (34.36)	825 (36.06)	237 (35.11)	0.6107
Women	2644 (64.57)	743 (65.64)	1463 (63.94)	438 (64.89)	
Education					
Less than high school	757 (18.49)	310 (27.39)	399 (17.44)	48 (7.11)	<0.0001
High school graduate/GED	734 (17.92)	246 (21.73)	397 (17.35)	91 (13.48)	
Attended vocational school/Trade school	2604 (63.59)	576 (50.88)	1492 (65.21)	536 (79.41)	
Occupation					
Management/Professional	1546 (37.75)	345 (20.48)	865 (37.81)	336 (49.78)	<0.0001
Other	2549 (62.25)	787 (69.52)	1423 (62.19)	339 (50.22)	
Alcohol use					
Yes	1864 (45.52)	470 (41.52)	1026 (44.84)	368 (54.52)	<0.0001
No	2231 (54.48)	662 (58.48)	1262 (55.16)	307 (45.48)	
Current Smoker					
Yes	3604 (88.20)	248 (21.95)	213 (9.33)	21 (3.12)	<0.0001
No	482 (11.80)	882 (78.05)	2069 (90.67)	653 (96.88)	
BMI, kg/m^2^	31.7 (7.1)	33.5 (6.45)	31.9 (7.3)	28.1 (6.4)	<0.0001
Blood Pressure, mmHg					
Systolic Blood Pressure	127 (16.71)	133 (16.39)	127 (15.94)	116 (14.54)	<0.0001
Diastolic Blood Pressure	75 (8.60)	76 (8.83)	76 (8.58)	73 (7.74)	<0.0001
Aldosterone (ng/dL) ^b^	4.30 (2.60, 7.10)	4.90 (2.90, 8.40)	4.30 (2.60, 7.00)	3.70 (2.20, 5.80)	<0.0001
eGFR (CKD-EPI) ml/min per 1.73 m^2 c^	85 (17.95)	83 (19.52)	85 (17.52)	91 (15.32)	<0.0001
Total Cholesterol, mg/dL	199 (39.40)	219 (39.34)	196 (37.46)	178 (30.20)	<0.0001
Fasting Plasma Glucose, mg/dL	100 (33.05)	114 (44.41)	97 (28.05)	86 (9.91)	<0.0001
Hemoglobin A1c (%)	5.9 (1.20)	6.5 (1.45)	5.8 (1.07)	5.2 (0.44)	<0.0001
Baseline Diabetes					
Yes	782 (19.10)	428 (37.81)	348 (15.21)	6 (0.89)	<0.0001
No	3313 (80.90)	704 (62.19)	1940 (84.79)	669 (99.11)	
Sodium (mmol/L)	140.56 (2.28)	140.59 (2.32)	140.63 (2.33)	140.29 (2.01)	0.0029
Potassium (mmol/L)	4.28 (0.44)	4.25 (0.46)	4.28 (0.44)	4.33 (0.34)	0.0015
Chloride (mmol/L)	104.3 (2.9)	103.85 (3.12)	104.4 (2.92)	104.74 (2.35)	<0.0001
Creatinine (mg/dL)	1 (0.53)	1.03 (0.66)	1 (0.5)	0.95 (0.38)	0.0059
Sodium Intake (mg)	2517.71 (1059.06)	2467.43 (1051.66)	2529.98 (1072.05)	2560.1 (1024.93)	0.1422
Potassium Intake (mg)	3898.07 (1802.17)	3775.73 (1770.06)	3912.71 (1812.82)	4052.78 (1807.97)	0.0059
Baseline individual cardiovascular health metrics	*n*				*P*-value ^e^
*N* (col%)		Poor (0–1)	Intermediate (2–3)	Ideal (4+)	
Smoking					
Poor	482	248 (21.91)	213 (9.31)	21 (3.11)	<0.0001
Intermediate	48	27 (2.39)	15 (0.66)	6 (0.89)	
Ideal	3565	857 (75.71)	2060 (90.03)	648 (96.00)	
BMI					
Poor	2165	733 (64.75)	1227 (53.63)	205 (30.37)	<0.0001
Intermediate	1360	380 (33.57)	781 (34.13)	199 (29.48)	
Ideal	570	19 (1.68)	280 (12.24)	271 (40.15)	
Physical Activity					
Poor	1963	734 (64.84)	1071 (46.81)	158 (23.41)	<0.0001
Intermediate	1336	383 (33.83)	770 (33.65)	183 (27.11)	
Ideal	796	15 (1.33)	447 (19.54)	334 (49.48)	
Dietary Intake					
Poor	2350	624 (55.12)	1337 (58.44)	389 (57.63)	<0.0001
Intermediate	1706	508 (44.88)	929 (40.60)	269 (39.85)	
Ideal	39	0 (0.00)	22 (0.96)	17 (2.52)	
Blood Pressure					
Poor	848	333 (29.42)	463 (20.24)	52 (7.70)	<0.0001
Intermediate	2415	783 (69.17)	1454 (63.55)	178 (26.37)	
Ideal	832	16 (1.41)	371 (16.22)	445 (65.93)	
Total Cholesterol					
Poor	602	300 (26.50)	277 (12.11)	25 (3.70)	<0.0001
Intermediate	1639	763 (67.40)	798 (34.88)	78 (11.56)	
Ideal	1854	69 (6.10)	1213 (53.02)	572 (84.74)	
Glucose					
Poor	666	369 (32.60)	292 (12.76)	5 (0.74)	<0.0001
Intermediate	1606	715 (63.16)	850 (37.15)	41 (6.07)	
Ideal	1823	48 (4.24)	1146 (50.09)	629 (93.19)	

^a^ Mean (Standard deviation) or Count (percentage) is listed. ^b^ Median (Q1, Q3) is used due to right skewed distribution of aldosterone. ^c^ EGFR CKD-EPI = Estimated glomerular filtration rate based on the Chronic Kidney Disease Epidemiology Collaboration. ^d^ To assess the differences of these variables across Life’s simple 7 categories, Chi-Square tests were used for categorical variables and one way ANOVAs were used for continuous variables except aldosterone. Kruskal–Wallis test was used for aldosterone due to a skewed distribution. ^e^ Fischer’s exact test was used.

**Table 2:** The association of categorical and continuous LS7 scores with aldosterone among all participants and stratified by age, sex, diabetes status, estimated Glomerular Filtration Rate (eGFR).

	Aldosterone (Median and IQR)	*n* (%) of Participants Taking ACE-Inhibitors, ARBs, Mineralocorticoid Receptor Antagonists and Statin Medications ^f^	Categorical LS7 Mean Ratio (95% CI)		Continuous LS7 Exp (ß)
			Intermediate vs. Poor	Ideal vs. poor	
All participants, *n* = 4095	4.30 (2.60, 7.10)	1294 (31.6)	0.85 (0.80, 0.90)	0.67 (0.61, 0.75)	0.89 (0.86, 0.91)
All participants, after adjusting for ACE-inhibitors, ARBs, mineralocorticoid receptor antagonists and statin medications			0.84 (0.78, 0.91)	0.61 (0.48, 0.78)	0.87 (0.84, 0.91)
Age < median ^a^, *n* = 2047	4.30 (2.60, 7.10)	404 (19.74)	0.68 (0.62, 0.74)	0.59 (0.53, 0.67)	0.85 (0.82, 0.88)
Age > median ^a^, *n* = 2048	4.30 (2.50, 7.30)	890 (43.46)	1.00 (0.93, 1.09)	0.72 (0.57, 0.91)	0.94 (0.90, 0.98)
Men ^b^, *n* = 1451	4.80 (3.00, 7.40) ^e^	427 (32.79)	0.97 (0.87, 1.08)	0.81 (0.69, 0.96)	0.95 (0.91, 0.98)
Women ^b^, *n* = 2644	4.10 (2.30, 7.00) ^e^	867 (29.43)	0.79 (0.74, 0.85)	0.61 (0.53, 0.70)	0.85 (0.82, 0.88)
Participants without diabetes ^c^, *n* = 3313	4.20 (2.50, 6.90) ^e^	820 (24.75)	0.82 (0.76, 0.88)	0.67 (0.59, 0.75)	0.88 (0.86, 0.92)
Participants with diabetes ^c^, *n* = 782	4.70 (2.90, 8.20) ^e^	474 (60.61)	0.99 (0.89, 1.10)	0.68 (0.21, 2.22)	0.95 (0.88, 1.03)
eGFR < median ^d^, *n* = 2048	5.00 (3.00, 8.00) ^e^	801 (39.11)	0.84 (0.78, 0.91)	0.64 (0.54, 0.75)	0.88 (0.85, 0.91)
eGFR > median ^d^, *n* = 2047	3.80 (2.20, 6.20) ^e^	493 (24.08)	0.84 (0.75, 0.93)	0.69 (0.59, 0.81)	0.90 (0.86, 0.93)

All analyses adjusted for age, sex, education, occupation, alcohol use and estimated glomerular filtration rate. ^a^ The *p*-values for the interaction of age with the association of categorical and continuous LS7 scores with aldosterone were *p* < 0.0001 and *p* = 0.0007, respectively. Median age was 55.5 (45.53, 64.59). ^b^ the *p*-values for the interaction of sex with the association of categorical and continuous LS7 scores with aldosterone were *p* = 0.0021 and *p* < 0.0001, respectively. ^c^ The *p*-values for the interaction of diabetes with the association of categorical and continuous LS7 scores with aldosterone were *p* = 0.0160 and *p* = 0.1231, respectively. ^d^ The *p*-values for the interaction of eGFR with the association of categorical and continuous LS7 scores with aldosterone were *p* < 0.0001 and *p* = 0.0007, respectively. Median eGFR was 95 (81.42, 109.32). ^e^ *p* < 0.0001 using wilcoxon rank sum test ^f^ *p*-values for differences in medication usage using wilcoxon rank sum test age *p* < 0.0001, sex *p* = 0.0268, diabetes *p* < 0.0001, eGFR *p* < 0.0001. ACE-inhibitors (Angiotensin Converting Enzyme), ARB (Angiotensin Receptor Blocker).

**Table 3:** The association of categorical and continuous LS7 scores with aldosterone among all participants and stratified by age, sex, diabetes status, estimated Glomerular Filtration Rate (eGFR) after excluding participants taking ACE inhibitors, ARBs, statins and mineralocorticoid receptor antagonists.

	Aldosterone (Median and IQR) ^e^	Categorical LS7 Mean Ratio (95% CI)		Continuous LS7 Exp (ß)
		Intermediate vs Poor	Ideal vs. poor	
All participants, *n* = 2801	4.10 (2.40, 6.80)	0.73 (0.67, 0.79)	0.62 (0.55, 0.70)	0.86 (0.83, 0.89)
Age < median ^a^, *n* = 1643	4.20 (2.40, 6.70)	0.81 (0.70, 0.93)	0.76 (0.65, 0.90)	0.93 (0.89, 0.97)
Age > median ^a^, *n* = 1158	4.10 (2.40, 6.80)	0.78 (0.71, 0.86)	0.60 (0.48, 0.74)	0.85 (0.81, 0.90)
Men ^b^, *n* = 1024	4.60 (2.90, 7.10)	0.91 (0.79, 1.05)	0.80 (0.67, 0.97)	0.94 (0.89, 0.98)
Women ^b^, *n* = 1777	3.90 (2.20, 6.60)	0.65 (0.59, 0.72)	0.54 (0.47, 0.63)	0.80 (0.76, 0.84)
Participants without diabetes ^c^, *n* =2493	4.00 (2.40, 6.60)	0.71 (0.65, 0.77)	0.64 (0.56, 0.72)	0.87 (0.84, 0.90)
Participants with diabetes ^c^, *n* = 308	4.60 (3.10, 8.60)	0.77 (0.63, 0.94)	0.51 (0.12, 2.10)	0.80 (0.70, 0.92)
eGFR < median ^d^, *n* = 1247	4.70 (2.90, 7.50)	0.80 (0.72, 0.89)	0.63 (0.53, 0.75)	0.87 (0.83, 0.91)
eGFR > median ^d^, *n* = 1554	3.70 (2.10, 6.00)	0.78 (0.68, 0.91)	0.68 (0.57, 0.81)	0.90 (0.86, 0.95)

^a^ The *p*-values for the interaction of age with the association of categorical and continuous LS7 scores with aldosterone were *p* = 0.1749 and *p* = 0.0164, respectively. ^b^ The *p*-values for the interaction of sex with the association of categorical and continuous LS7 scores with aldosterone were *p* < 0.0001 and *p* < 0.0001, respectively. ^c^ The *p*-values for the interaction of diabetes with the association of categorical and continuous LS7 scores with aldosterone were *p* = 0.2712 and *p* = 0.6031, respectively. ^d^ The *p*-values for the interaction of eGFR with the association of categorical and continuous LS7 scores with aldosterone were *p* = 0.6665 and *p* = 0.2829, respectively. ^e^ *p*-values for differences in median aldosterone using wilcoxon rank sum test, age *p* = 0.9294, sex *p* < 0.0001, diabetes *p* < 0.0001, eGFR *p* < 0.0001.

**Table 4:** Associations of individual Life’s Simple 7 metrics with aldosterone.

Life’s Simple 7 Metrics	Mean Ratios (95% CI)			
	Intermediate vs. Poor	*p*-Value	Ideal vs. Poor	*p*-Value
Smoking	0.76 (0.57,1.01)	0.0554	0.71 (0.65,0.76)	<0.0001
Body Mass Index	0.96 (0.90, 1.02)	0.1968	0.83 (0.75, 0.92)	0.0003
Physical Activity	0.95 (0.89, 1.02)	0.1497	1.04 (0.96, 1.12)	0.2992
Diet	1.01 (0.96, 1.08)	0.6202	0.72 (0.49, 1.06)	0.0938
Blood pressure	1.04 (0.97, 1.11)	0.3337	0.77 (0.69, 0.86)	<0.0001
Total Cholesterol	1.04 (0.96, 1.12)	0.3656	0.89 (0.82, 0.97)	0.0090
Fasting Plasma Glucose	0.84 (0.78, 0.91)	<0.0001	0.78 (0.72, 0.84)	<0.0001

Adjusted for age, sex, education, occupation, alcohol use and eGFR. *p* < 0.05 indicates at least one comparison was significant.

## Supplementary Materials

The following are available online at https://www.mdpi.com/2072-6643/11/5/955/s1, Table S1: Baseline characteristics comparison between excluded and included participants, Table S2: Definitions of Poor, Intermediate, Ideal Cardiovascular Health, Table S3: The Association of Categorical and Continuous LS7 Scores with Aldosterone among All Participants (excluding participants with aldosterone below the lower limit) and Stratified by Age, Sex, Diabetes Status, Estimated Glomerular Filtration Rate (eGFR), Table S4: The association of Life’s Simple 7 with Aldosterone, (A) Median aldosterone levels across three categories of total LS7 scores using 0–14 scoring system and (B) The association of categorical and continuous LS7 scores (using 0–14 scoring system )with aldosterone among all participants, Table S5: Association of Individual LS7 Metrics with Aldosterone stratified by Sex among All Participants and after excluding participants on medications altering aldosterone.
