# Supplementary material for: The Association of Life’s Simple 7 with Aldosterone among African Americans in the Jackson Heart Study
Source: Nutrients. 2019 Apr 26;11(5):955. doi: 10.3390/nu11050955 (PMC6566676; doi:10.3390/nu11050955)
Supplement: Supplementary file 1 [file nutrients-11-00955-s001.pdf]

Table S1. Baseline characteristics comparison between excluded and included participants.

Variable [a]	Level	Excluded (N = 1211)	Included (N = 4095)	p-value
Age		55.7 (13.65)	55.24 (12.62)	0.2717
Sex	Women	727 (60.03)	2644 (64.57)	0.0040
	Men	484 (39.97)	1451 (35.43)	
Education	Less than high school	319 (26.9)	757 (18.49)	<0.0001
	High school graduate / GED	218 (18.38)	734 (17.92)	
	Attended vocational school /Trade school	649 (54.72)	2604 (63.59)	
Occupation	Management/Professional	332 (27.42)	1546 (37.75)	<0.0001
	Other	879 (72.58)	2549 (62.25)	
Alcohol use	No	626 (53.01)	2231 (54.48)	0.3701
	Yes	555 (46.99)	1864 (45.52)	
Current Smoker	No	961 (82)	3604 (88.2)	<0.0001
	Yes	211 (18)	482 (11.8)	
BMI, kg/m^2		31.78 (7.45)	31.74 (7.18)	0.8918
Blood Pressure, mmHg	Systolic Blood Pressure	127.66 (17.55)	127.43 (16.71)	0.6908
	Diastolic Blood Pressure	74.94 (9.33)	75.93 (8.6)	0.0006
Aldosterone (ng/dL) [b]		5.8 (4.45)	5.81 (5.82)	0.9610
eGFR [c] (CKD-EPI) ml/min per 1.73 m^2		85.99 (20.66)	85.85 (17.95)	0.8231
Total Cholesterol, mg/dL		196.4 (43.83)	199.83 (39.4)	0.0342
Fasting Plasma Glucose, mg/dL		100.02 (35.08)	100.05 (33.05)	0.9853
Hemoglobin A1c (%)		6.25 (1.56)	5.91 (1.2)	<0.0001
Baseline Diabetes	No	775 (67.69)	3313 (80.9)	<0.0001
	Yes	370 (32.31)	782 (19.1)	
Smoking	Poor	211 (18.79)	482 (11.77)	<0.0001
	Intermediate	16 (1.42)	48 (1.17)	
	Ideal	896 (79.79)	3565 (87.06)	
Body Mass Index	Poor	657 (54.89)	2165 (52.87)	0.0027
	Intermediate	341 (28.49)	1360 (33.21)	
	Ideal	199 (16.62)	570 (13.92)	
Physical Activity	Poor	652 (54.06)	1963 (47.94)	0.0007
	Intermediate	337 (27.94)	1336 (32.63)	
	Ideal	217 (17.99)	796 (19.44)	
Dietary Intake	Poor	331 (49.7)	2350 (57.39)	0.0005
	Intermediate	324 (48.65)	1706 (41.66)	
	Ideal	11 (1.65)	39 (0.95)	
Blood Pressure	Poor	267 (23.2)	848 (20.71)	0.0117
	Intermediate	692 (60.12)	2415 (58.97)	
	Ideal	192 (16.68)	832 (20.32)	
Total Cholesterol	Poor	104 (15.83)	602 (14.7)	0.0718
	Intermediate	232 (35.31)	1639 (40.02)	
	Ideal	321 (48.86)	1854 (45.27)	
Fasting Plasma Glucose	Poor	289 (32.36)	666 (16.26)	<0.0001
	Intermediate	350 (39.19)	1606 (39.22)	
	Ideal	254 (28.44)	1823 (44.52)	

[a] Mean (Standard deviation) or Count (percentage) is listed. [b] Median (Q1, Q3) is used due to right skewed distribution of aldosterone. [c] EGFR CKD-EPI = Estimated glomerular filtration rate based on the Chronic Kidney Disease Epidemiology Collaboration.

Table S2. Definitions of Poor, Intermediate, Ideal Cardiovascular Health [a].

Goal/Metric	Poor health	Intermediate health	Ideal health
Current smoking	Yes	Former ≤ 12 months	Never or quit ≥12 months
Total cholesterol	≥240 mg/dL	200–239 mg/dl or treated to goal	<200 mg/dl
Blood pressure	SBP≥140 or DBP ≥90	SBP 120-139 or DBP 80-89 or treated to goal	<120/<80 mm Hg
Fasting plasma glucose	≥126 mg/dL	100–125 mg/dl or treated to goal	<100 mg/dL
Body mass index	≥30 kg/m^2	25–29.9 kg/m^2	<25 kg/m^2
Physical activity	None	1-149 min/week moderate intensity or 1-74 min/week vigorous intensity	≥150 min/week moderate intensity or ≥75 min/week vigorous intensity
Healthy diet score [b]	0-1 components	2-3 components	4–5 components
Points for LS7 Score per Metric	0	0	1

[a] Adapted from The American Heart Association's Strategic Planning Task Force and Statistical Committee 2020 Guidelines[1]. [b] Adapted for JHS: Fruits and vegetables ≥4.5 cups/day, non-fried fish ≥two 3.5 ounce servings per week, fiber-rich whole grains ≥ three 1 ounce servings/day, sodium <1500 mg/day, sugar-sweetened beverages ≤ 36 ounces/week.

Table S3. The Association of Categorical and Continuous LS7 Scores with Aldosterone among All Participants(excluding participants with aldosterone below the lower limit) and Stratified by Age, Sex, Diabetes Status, Estimated Glomerular Filtration Rate (eGFR).

	Aldosterone (median and IQR)	n (%) of participants taking ACE-inhibitors, ARBs, mineralocorticoid receptor antagonists and statin medications [f]	Categorical LS7 Mean ratio (95% CI)		Continuous LS7 Exp(ß)
			Intermediate vs Poor	Ideal vs. poor	
All participants, n = 3382	5.10 (3.40, 8.00)	1110 (32.82)	0.87 (0.82, 0.93)	0.71 (0.63, 0.79)	0.90 (0.88, 0.93)
All participants, after adjusting for ACE-inhibitors, ARBs, mineralocorticoid receptor antagonists and statin medications			0.83 (0.77, 0.91)	0.60 (0.47, 0.77)	0.87 (0.83, 0.91)
Age <median [a], n = 1698	5.00 (3.40, 7.70)	357 (21.02)	0.70 (0.64, 0.77)	0.63 (0.55, 0.71)	0.87 (0.84, 0.90)
Age >median [a], n = 1684	5.20 (3.40, 8.20)	753 (44.71)	1.03 (0.94, 1.12)	0.78 (0.62, 0.98)	0.96 (0.92, 1.00)
Men [b], n = 1270	5.30 (3.60, 8.10) [e]	383 (30.16)	0.97 (0.87, 1.08)	0.83 (0.70, 0.99)	0.95 (0.91, 0.99)

Women [b], *n* = 2112	5.00 (3.30, 8.00) [e]	727 (34.42)	0.83 (0.77, 0.89)	0.65 (0.56, 0.75)	0.87 (0.84, 0.90)
Participants without diabetes [c], *n* = 2712	5.00 (3.40, 7.70) [e]	706 (26.03)	0.84 (0.78, 0.91)	0.70 (0.63, 0.79)	0.90 (0.88, 0.93)
Participants with diabetes [c], *n* = 670	5.50 (3.60, 9.00) [e]	404 (60.30)	1.00 (0.90, 1.12)	0.67 (0.20, 2.31)	0.95 (0.88, 1.03)
eGFR <median [d], *n* = 1783	5.50 (3.70, 8.70) [e]	711 (39.88)	0.87 (0.80, 0.95)	0.67 (0.56, 0.79)	0.89 (0.86, 0.93)
eGFR >median [d], *n* = 1599	4.70 (3.20, 7.20) [e]	399 (24.95)	0.86 (0.77, 0.96)	0.73 (0.62, 0.85)	0.90 (0.87, 0.95)

All analyses adjusted for age, sex, education, occupation, alcohol use and estimated glomerular filtration rate. [a] The p-values for the interaction of age with the association of categorical and continuous LS7 scores with aldosterone were p<0.0001 and $p = 0.0006$, respectively. [b] the p-values for the interaction of sex with the association of categorical and continuous LS7 scores with aldosterone were p=0.0205 and $p = 0.0013$, respectively. [c] The p-values for the interaction of diabetes with the association of categorical and continuous LS7 scores with aldosterone were p=0.0460 and $p = 0.2456$, respectively. [d] The p-values for the interaction of eGFR with the association of categorical and continuous LS7 scores with aldosterone were p=0.6494 and $p = 0.5243$, respectively. [e] p<0.0001 using wilcoxon rank sum test [f] P-values for differences in medication usage using wilcoxon rank sum test age $p < 0.0001$, sex $p = 0.0268$, diabetes $p < 0.0001$, eGFR $p < 0.0001$ [a] Median age was 55.5(45.44, 64.64) [d] Median eGFR was 94.04 (80.46, 107.55) ACE-inhibitors (Angiotensin Converting Enzyme), ARB (Angiotensin Receptor Blocker).

Table S4. The association of Life's Simple 7 with Aldosterone, (A) Median aldosterone levels across three categories of total LS7 scores using 0–14 scoring system and (B) the association of categorical and continuous LS7 scores (using 0-14 scoring system [a]) with aldosterone among all participants.

Table S4A.

	Inadequate (*n* = 420)	Average (*n* = 3119)	Optimal (*n* = 556)	*p*-value [b]
Aldosterone (ng/dL)	5.00 (3.10, 8.20)	4.40 (2.60, 7.30)	3.75 (2.30, 5.80)	<0.0001

Table S4B.

All participants	Average vs Inadequate	Optimal vs.Inadequate	Continuous LS7 Exp(ß)
Mean ratio (95% CI)	0.91 (0.84, 1.00)	0.72 (0.64, 0.83)	0.94 (0.93, 0.96)

[a] Analysis using 0-14 scoring system with poor ==0, intermediate ==1 and ideal==2, inadequate (0–4 points), average (5–9 points), and optimal (10–14 points) [b] Wilcoxon rank sum test used.

Table S5. Association of Individual LS7 Metrics with Aldosterone stratified by Sex among All Participants and after excluding participants on medications altering aldosterone.

Among all participants	Among participants after excluding subjects on aldosterone altering medications

Individual LS7 metrics	Sex	*p* –value [c]	Intermediate vs Poor[a]	Ideal vs Poor [a]	*p*-value [c]	Intermediate vs Poor [b]	Ideal vs Poor [b]
Smoking	Men	<0.0001	1.07 (0.73, 1.59)	0.96 (0.83, 1.10)	<0.0001	1.15 (0.72, 1.83)	0.92 (0.78, 1.08)
	Women		0.59 (0.39, 0.89)	0.58 (0.53, 0.64)		0.53 (0.30, 0.96)	0.48 (0.44, 0.53)
Body Mass Index	Men	0.0001	0.81 (0.73, 0.90)	0.77 (0.6, 0.90)	0.0320	0.87 (0.76, 0.98)	0.82 (0.68, 0.98)
	Women		1.06 (0.99, 1.15)	0.86 (0.76, 0.98)		1.06 (0.96, 1.16)	0.80 (0.68, 0.94)
Physical activity	Men	0.0392	1.07 (0.96, 1.20)	1.09 (0.96, 1.23)	0.0352	1.05 (0.92, 1.21)	0.97 (0.83, 1.130
	Women		0.89 (0.82, 0.97)	1.02 (0.93, 1.12)		0.86 (0.77, 0.95)	1.00 (0.89, 1.13)
Diet	Men	0.0342	1.12 (1.02, 1.23)	1.01 (0.54, 1.86)	0.0897	1.06 (0.94, 1.20)	1.08 (0.50, 2.32)
	Women		0.96 (0.90, 1.04)	0.64 (0.40, 1.03)		0.91 (0.83, 1.00)	0.53 (0.23, 1.22)
Blood Pressure	Men	0.1188	1.01 (0.90, 1.13)	0.85 (0.72, 1.01)	0.0196	0.99 (0.86, 1.15)	0.90 (0.75, 1.08)
	Women		1.05 (0.96, 1.15)	0.73 (0.63, 0.84)		1.18 (1.04, 1.34)	0.83 (0.70, 0.98)
Total Cholesterol	Men	0.0746	1.10 (0.94, 1.28)	1.01 (0.87, 1.18)	0.0746	1.03 (0.86, 1.24)	0.99 (0.83, 1.18)
	Women		1.02 (0.92, 1.12)	0.84 (0.76, 0.93)		1.26 (1.10, 1.44)	0.93 (0.81, 1.06)
Glucose	Men	0.5520	0.85 (0.75, 0.97)	0.81 (0.71, 0.93)	0.5520	0.81 (0.68, 0.97)	0.78 (0.65, 0.93)
	Women		0.84 (0.77, 0.92)	0.76 (0.69, 0.83)		0.77 (0.68, 0.88)	0.73 (0.64, 0.83)

[a] Among all participants [b] among participants after excluding aldosterone altering medications [c] *p*-value obtained using Wilcoxon rank sum test.
